# Advances in Biodegradable Soft Robots

**DOI:** 10.3390/polym14214574

**Published:** 2022-10-28

**Authors:** Jiwon Kim, Harim Park, ChangKyu Yoon

**Affiliations:** 1 Department of Mechanical Systems Engineering, Sookmyung Women’s University, Seoul 04310, Korea; 2 Institute of Advanced Materials and Systems, Sookmyung Women’s University, Seoul 04310, Korea

**Keywords:** stimuli-responsive materials, hybrid actuators, intelligent systems

## Abstract

Biodegradable soft robots have been proposed for a variety of intelligent applications in soft robotics, flexible electronics, and bionics. Biodegradability offers an extraordinary functional advantage to soft robots for operations accompanying smart shape transformation in response to external stimuli such as heat, pH, and light. This review primarily surveyed the current advanced scientific and engineering strategies for integrating biodegradable materials within stimuli-responsive soft robots. It also focused on the fabrication methodologies of multiscale biodegradable soft robots, and highlighted the role of biodegradable soft robots in enhancing the multifunctional properties of drug delivery capsules, biopsy tools, smart actuators, and sensors. Lastly, the current challenges and perspectives on the future development of intelligent soft robots for operation in real environments were discussed.

## 1. Introduction

Multiscale stimuli-responsive hydrogel-based soft robots have demonstrated a variety of intelligent applications in manipulators, wearable electronics, and healthcare systems [1,2,3,4,5,6,7]. In general, stimuli-responsive soft robots have components made of elastic materials and actuation is achieved through continuum material deformations [8,9,10,11,12,13]. Compared with rigid body systems, stimuli-responsive soft robots exhibit a high degree of continuous shape deformation when triggered by external stimuli such as pneumatics, heat, pH, light, or even biomaterials [7,14,15]. Most soft robots are composed of stimuli-responsive hydrogel network systems (e.g., *N*-isopropyl acrylamide (NIPAM)), which exhibit large swelling/deswelling in aqueous environments, owing to their inherent porous nature [16]. NIPAM-based hydrogels, in particular, exhibit unique physicochemical thermal property changes between 32 and 36 °C by adjusting the lower critical solution temperature (LCST) [16,17]. In addition, more recent works have extensively hybridized multi-functional additives (e.g., graphene, nanowires, and liquid crystals) with stimuli-responsive hydrogel networks to develop multi-functional intelligent soft robots with enhanced mechanical, electrical, and/or optical properties [18].

From another perspective of stimuli-responsive material selection and design, patterning or structuring techniques constitute a critical aspect for precisely manufacturing hydrogel-based soft robots. Some popular methods for creating 3D systems include top-down approaches such as photolithography, electron beam, and replica patterning using conventional thin-film additives and subtractive fabrication techniques [5,8,19,20]. Furthermore, self-folding is an emerging innovative method to design 3D structures. The self-folding approach mainly utilizes an out-of-place deformation associated with photo-patterned 2D thin film structures, which turn into 3D folded, curved, or rolled shapes upon encountering external triggers such as heat, pH, and light without any manual control [8,21]. This combination of self-folding and photolithographic strategies has been utilized to develop intelligent soft robotic applications including actuators and sensors [22,23]. In addition, 3D/4D printing has been highlighted as an innovative new technology for patterning 3D structures and their time-dependent shape changes with an appropriate external trigger [24,25,26,27]. The combination of stimuli-responsive materials and 3D/4D printing techniques has also provided a new direction to the development of smart soft robot design and operation.

**Figure 1:**
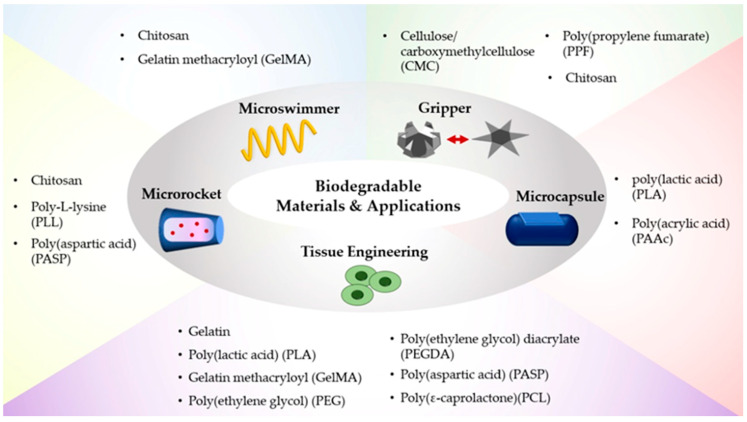
Biodegradable materials and their applications.

Meanwhile, extensive comprehensive reviews and discussions of stimuli-responsive materials, and their fabrications and applications have been presented [1,2,7]. More recently, integrated hybrid stimuli-responsive hydrogel systems were actively highlighted for a more comprehensive analysis of specific soft robotic viewpoints [2,3,4,6,18,28,29]; however, most of them rarely discussed biodegradable soft robots. Addressing the same, this review surveyed the recent advances in biodegradable soft robots. First, this review focused on the biodegradable materials utilized in soft robotics that exhibit multi-functional properties in response to stimuli such as heat, pH, light, and biomaterials (Section 2. Biodegradable Materials for Soft Robots). Next, we categorized the various fabrication methods such as 3D/4D printing and photolithography to structure biodegradable soft robots accompanied by external, stimuli-driven, smart shape transformation (Section 3. Fabrication Methods for Soft Robots). Further, we discussed the diverse applications of biodegradable soft robots in the form of actuators, sensors, drug delivery capsules, biopsies, etc. (Section 4. Applications of Biodegradable Soft Robots). Lastly, we highlighted the current challenges of biodegradable and stimuli-responsive soft robots and emphasized perspectives on the future development of intelligent, multiscale, tethered/untethered soft robots for their application in real environments (Section 5. Conclusion and Outlook). The overall, comprehensive, biodegradable materials and their applications are schematically described in Figure 1.

## 2. Biodegradable Materials for Soft Robots

The variety of biodegradable materials described in Table 1 can be utilized to create intelligent soft robots which can degrade after accomplishing their specified mechanical locomotion and function in the fields of targeted drug delivery, microsurgery, localized diagnosis, and smart actuators [24]. Biodegradable materials are mainly synthesized in the form of polymers, which are categorized as natural and artificial soft matter [30,31]. Among the many naturally derived biodegradable materials, protein-based polymers, chitosan, cellulose, and gelatin are the most utilized natural polymers to construct intelligent soft robots such as helical-shaped, small-scale swimmers and grippers [32,33]. Chitosan is a natural cationic polymer obtained by the deacetylation of chitin, which is insoluble in water, and alkaline and dissoluble in acidic solutions [34]. Specifically, chitosan can be enzymatically degraded by lysozyme and chitosanase enzymes [35]. These biodegradable properties of chitosan make it a promising candidate in targeted drug/cell delivery in the form of a 3D micro-swimmer in water [32]. In addition, cellulose/carboxymethylcellulose (CMC) is an anionic, water-soluble cellulose polymer formed by the reaction between alkali and chloroacetic acid [36]. CMC exhibits unique chemical and physical properties such as biocompatibility, biodegradability, softness, transparency, high viscosity at low concentrations, and swelling at high pH [32,37]. Furthermore, gelatin is another natural protein obtained by either acid or alkaline hydrolysis of a collagen based on anionic and cationic groups in a gel network, with low gelation temperature (e.g., spray-dried goat skin gelatin, freeze-dried goat skin gelatin, and commercial bovine gelatin: 22.4–25.2 °C [38,39]). Gelatin has diverse advantages, including non-toxicity, high water absorption, biocompatibility, and biodegradability, and is applicable in a variety of biomedical healthcare systems [40,41,42,43].

Lactic acid (LA, 2-hydroxypropionic acid, CH_3_CHOHCOOH), a naturally occurring organic acid, exists in two enantiomeric forms: L- and D-LA. LA is a building block of poly(lactic acid) (PLA) [44]. PLA is a thermoplastic aliphatic polyester derived from renewable plant sources such as starch and sugar [45]. It is biocompatible with the human body [46] and is easily degraded by the hydrolysis of ester bonds without requiring any enzymes [44]. Owing to its simple degradation process and biocompatibility, PLA has been widely utilized in diverse biomedical applications [47,48,49,50]. By chemically tuning the L and D isomers of LA, LA can also be polymerized into a variety of poly-L-LA (PLLA), pure poly-D-LA (PDLA), and poly-D,L-LA (PDLLA) [44], known as the isoforms of PLA [51]. These LA-based hydrogels are biocompatible and biodegradable. However, PDLA and PDLLA, upon degradation, produce D-lactic acid, which is slightly harmful to the human body [45]. In comparison, PLLA produces L-lactic acid, which is harmless to the human body [52]. Although PLA and its isoforms have many advantages, they have some limitations: (1) low degradation rate; (2) hydrophobicity; and (3) low impact toughness associated with their use [44]. To overcome these limitations, different physical blends of polymers [53] with the addition of moieties [54,55] have been widely utilized.

Poly-L-lysine (PLL) polymer, composed of lysine amino acids, is hydrophilic, biocompatible, biodegradable [56], and a polypeptide isomer of polylysine [57]. Since PLL is a biocompatible, biodegradable, and hydrophilic polypeptide [56], it is used in various biomedical applications [58,59,60,61], especially gene delivery [59,62]. The repeating units of PLL carry a positive charge on the ε-amine side chain at a physiological pH (≈7.4). Therefore, PLL can concentrate plasmid DNA to varying degrees depending on the salt concentration [59]. In addition, the gene delivery efficiency of PLL depends on its molecular weight [62], increasing with it. However, its cytotoxicity also increases with its molecular weight [62]. Furthermore, an increase in the PLL length increases the cytotoxicity of PLL [59]. Because of this problem, PLL modification is necessary to tune the properties of PLL [59]. One example of PLL modification to enhance the efficacy of gene delivery is chemical modification, which is implemented by conjugating ligands, such as asialoorosomucoid, transferrin, folate, monoclonal antibodies, and basic fibroblast growth factors with PLL [62].

Based on a different perspective of artificial biodegradable materials, gelatin methacryloyl (GelMA) is a synthetic, gelatin-based, biodegradable polymer, which is chemically modified with methacrylic anhydride (MAA) [63]. Generally, GelMA supports good cell attachment and growth, and is gradually degraded by cell-released enzymes during the culture process [64]. From the viewpoint of GelMA-based soft robots, the degree of methacryloylation and its concentration are key factors in providing effective manufacturability, functionality, and degradability [64,65,66,67]. Furthermore, owing to its low mechanical strength (~50 to 150 KPa) [63], short degradation time (~7 to 14 days) [63], and high swelling ratio [68], the gelation and operation times of GelMA-based soft robots are essential to avoid degradation and inflammation in tissue engineering [69].

Poly(ethylene glycol) (PEG) is another synthetic, biodegradable polymer suitable for undergoing limited metabolism in a physiological environment and exhibits excellent biocompatibility, including non-ionic and low inflammation [78]. Particularly, to enhance the mechanical properties of PEG, poly(ethylene glycol) diacrylate (PEGDA) can be synthesized by combining PEG with acryloyl chloride [79]. PEGDA has a higher shear storage modulus (e.g., 68 KPa at 20 wt% [79]) than PEG (e.g., 13.7 KPa at 20 wt% Pluronic [79]); therefore, the mechanical stability of PEGDA-based soft robots is higher than its PEG-based counterparts. However, PEGDA degrades slowly in vivo, so it is not suitable for long-term implantable applications [81]. Lately, PEG- or PEGDA-based soft robots have been extensively utilized as biodegradable microrobots [82,97,98], as micro-swimmers [82,99] for targeted therapeutic healthcare applications and tissue engineering [80].

Poly(propylene fumarate) (PPF) is a biodegradable and non-swellable, segmented polymer [89]. Similar to PLA, PPF is also an aliphatic polyester, which degrades via the hydrolysis of its ester bonds [83], and is affected by the molecular mass of the backbone chain, types of cross-linkers, and cross-linking density [100,101,102]. PPF is also biocompatible and non-toxic, confirmed by the cellular cytotoxicity standards (ISO 10993-5) [83,84,85,86,87]. Thus, PPF-based soft robots have been proposed as photolithographically patterned, self-folding healthcare theragrippers [88] and stimuli-responsive grippers [89]. In addition, poly(aspartic acid) (PASP) is a water-soluble and pH-responsive biodegradable polymer [103,104]. In general, PASP is a smooth, intact, and robust material that cannot be destroyed by an organic solvent, acid, or base solution [90]. PASP has various side-chain functional groups (e.g., amino, carboxyl, and hydroxyl) [90]. Owing to the carboxyl group in PASP, electrostatic interactions can occur with the amino groups of other materials [90]. These interactions facilitate the bonding of PASP with materials bearing amino groups, such that PASP-based micro-composites can serve as drug delivery microcarriers [90]. Despite this advantage, PASP has a limitation: the synthesis of PASP-based hydrogels is relatively more complicated than that of other anionic hydrogels [91].

Moreover, poly(acrylic acid) (PAAc) is a water-soluble, biodegradable, and high-molecular-weight polymer that is polymerized by the monomer, acrylic acid [92]. In particular, the carboxylic acid in the PAAc network makes it suitable for manufacturing a pH-responsive drug delivery system [92]. This unique characteristic of pH-responsive PAAc, combined with polyacrylamide (PAAm), provides a lipophilic drug delivery microrobot, which can be utilized in a wide range of pH such as in the stomach (pH = 2) and intestines (pH = 8) [93]. Poly(ε-caprolactone) or polycaprolactone (PCL) is another biodegradable semi-crystalline polymer, which is semi-rigid at room temperature [79,94,95]. Normally, PCL is degraded by enzymes or fungi for 1 to 2 years and has a relatively high stiffness with an elastic modulus of ~0.21 to 0.44 GPa compared to other biodegradable materials [95]. To improve its degradation rate and mechanical properties, PCL is blended with lactic acids (e.g., PLA, PLLA, PLGA, and polyethers) [79,105]. Moreover, it has recently been noted that PCL has a high tensile strength (~23 MPa) and elongation before breaking (more than 4700%) [79].

## 3. Fabrication Methods for Soft Robots

After tailoring biodegradable materials, suitable fabrication techniques must be developed to manufacture biodegradable 3D soft robots. Various fabrication strategies such as photolithography and 3D/4D printing have been employed to manufacture biodegradable 3D soft robots.

### 3.1. Photolithography

**Figure 2:**
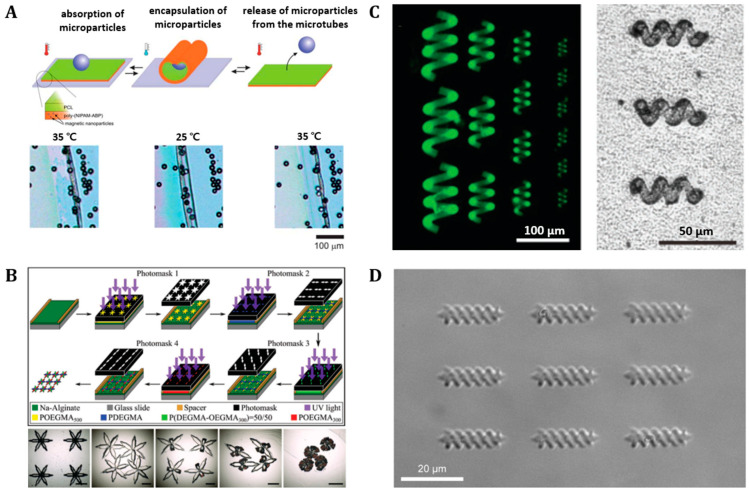
**Microscale biorobots fabricated by photolithography and two photon polymerization processes.** (**A**) **Photolithography**: Scheme of the capture and release of microparticles by self-rolling microtubes (upper) and images of the encapsulation and release of microparticles from microtubes at different temperatures (lower). Reproduced with permission [107]. Copyright 2010, The Royal Society of Chemistry. (**B**) **Photolithography**: Schematic representation of the fabrication process for four-state, thermally responsive grippers (upper) and representative images of grippers (lower). Reproduced with permission [108]. Copyright 2018, Wiley-VCH. (**C**) **Two-photon lithography**: Fluorescent image of helical microstructures with different sizes (left) and optical image of helical micro-swimmers decorated with magnetic nanoparticles (right). Reproduced with permission [64]. Copyright 2018, Wiley-VCH. (**D**) **Two-photon lithography**: Optical microscopy image of 3 × 3 array of the micro-swimmers. Reproduced with permission [32]. Copyright 2018, The American Chemical Society.

Several innovative fabrication methods have been proposed to construct multiscale, complex 3D structures, including additive and subtractive, process-based, lithographic approaches (e.g., photo [106,107,108], two-photon [32,64], and electron beam [109,110]). In particular, lithographic patterning technique is highly parallel and precise for manufacturing micro- and nano-semiconductor chips [111]. The photolithographic patterning process generally involves transferring the designed pattern from the mask or reticle to the photoresist on the wafer surface [111]. However, photolithographic techniques involve several two-dimensional (2D), planar, additive and subtractive, serial deposition and removal processes. Thus, complex and shape-changing 3D soft robots can be manufactured by utilizing a combined photolithography and self-folding strategy, which subjects a 2D thin film to 3D bent, curved, rolled, or folded shape changes without any manual control [107,108]. For example, Zakharchenko et al. have proposed thermoresponsive, shape-transformable, and partially biodegradable bilayer microtubes composed of poly(N-isopropylacrylamide) copolymer, containing 1 mol% of 4-acryloylbenzophenone comonomer (poly(NIPAM-ABP)) and polycaprolactone (PCL) (Figure 2A) [107]. In addition, Kobayashi et al. fabricated fully biodegradable (poly[oligo (ethylene glycol) methylether methacrylate] (POEGMA) and poly(di(ethylene glycol) methyl ether methacrylate) (PDEGMA) soft robots using a combined self-folding and photolithography strategy (Figure 2B) [108]. Photolithographic bilayering or panel-hinge patterning, comprising active and passive stimuli-responsive properties, have been primarily selected for converting 2D thin films into self-folded (e.g., self-curved, -rolled, -bent, and -twisted) 3D structures [88,112,113]. 

More recently, to create more complex micro- or nanoscale 3D structures, advanced fabrication technologies, such as two-photon polymerization (TPP) and two-photon lithography (TPL), have been widely developed [64,114,115,116]. TPP, also known as direct laser writing (DLW), has high spatial resolution and ultra-precision in micro- and nanoscale fabrication [104]. In short, TPP is a layer-by-layer method, which, unlike lithographic patterning which requires a mask, does not require the need to use a mask to fabricate complex structures [116]. Furthermore, different from conventional single-photon polymerization, TPP allows the photoinitiator (PI) molecule contained in the polymerization resist to absorb two photons and cause polymerization in a highly localized area [116]. This method has advantages in manufacturing multiscale 3D micro- or nanostructures of various materials such as polymers or hybrid metals with a subdiffraction-limit resolution (<100 nm) [114]. Specifically, TPP-driven, helical-shaped, microscale soft robots have been extensively developed as non-invasive biomedical devices (e.g., gelatin methacryloyl (GelMA)-based biodegradable micro-swimmers (Figure 2C) [64], and chitosan drug delivery micro-swimmers (Figure 2D) [32]).

### 3.2. 3D/4D Printing

**Figure 3:**
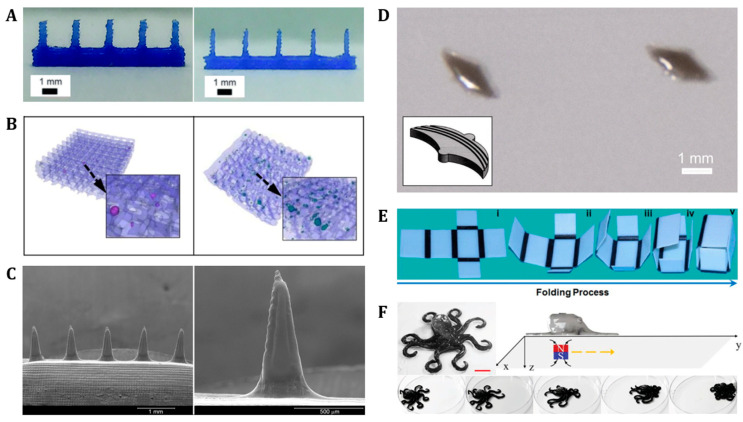
**Biomedical applications fabricated by 3D/4D printing**. (**A**) **3D Extrusion:** Optical images of microneedles fabricated by FDM. Reproduced with permission [118]. Copyright 2018, The Royal Society of Chemistry. (**B**) **3D Extrusion:** Scaffolds fabricated by 3D plotting. Reproduced with permission [120]. Copyright 2016, Wiley Periodicals. (**C**) **3D Jetting:** Miconazole-loaded Gantrez AN 169 BF microneedle array fabricated by Inkjet. Reproduced with permission [123]. Copyright 2014, Elsevier. (**D**) **3D Jetting:** Bat-shaped polymer structure printed by a PolyJet 3D printer. Reproduced with permission [126]. Copyright 2014, The American Society of Mechanical Engineers. (**E**) **4D printing:** A self-folding and -opening box. Reproduced with permission [26]. Copyright 2013, AIP publishing. (**F**) **4D printing:** Octopus-shaped soft robot exhibiting a forward movement. Reproduced with permission [128]. Copyright 2019, Wiley-VCH.

3D printing methods have also been widely utilized for manufacturing biodegradable soft robots applicable in biomedical engineering fields, including targeted drug delivery, biopsy, and tissue engineering [78,117]. In general, 3D printing techniques such as fused deposition modeling (FDM) [117,118,119], 3D plotting [78,120], inkjet [78,121,122,123,124], and PolyJet [125,126] possess the advantages of a high resolution and accuracy for pattern structures with automatically programmed geometry and repeatability. Among the several 3D printing techniques, FDM is most widely used [127]. FDM is a type of extrusion fabrication, which uses thermoplastic polymers in the form of filaments. To print a 3D structure directly, the filaments are melted in a nozzle, and the melted material is extruded to deposit onto the build platform with repeated, layer-by-layer processes until the layers fuse and solidify [117,119]. For example, Figure 3A shows biodegradable polymer microneedles for transdermal drug delivery printed using the FDM method. This FDM-based 3D microneedle pattern was designed using 1 to 55 µm printing tip sizes, which successfully broke into porcine skin [118]. As another example, bone tissue engineering scaffolds can be precisely patterned using the FDM strategy. Specifically, poly(D,L-lactide:glycolide) (DL-PLGA) and β-tricalcium phosphate (β-TCP) nanocomposites have been FDM-based 3D printed with hydroxyapatite (HA) coating on surfaces [119]. In addition, 3D plotting is another FDM-based 3D printing technique that extrudes viscous materials such as liquids or pastes [78]. For a specific example, Dávila et al. fabricated a biodegradable PCL/β-TCP scaffold, with improved hydrophilic cell adhesion and compressive strength, via 3D mini-screw extrusion printing, based on FDM printing [120]. The advantage of 3D mini-screw printing is that it can program diverse ratios of compositions of materials during 3D printing which simultaneously display different mechanical, chemical, and physical properties (Figure 3B) [120].

Furthermore, inkjet printing can be classified into continuous and drop-on-demand (DOD) systems [78,121,122]. In a continuous ejection system, the pressure of the print head is controlled, and the nozzle continuously generates jets. The jet then breaks into droplets of a uniform size and spacing. The DOD system differs from the continuous system in that it ejects ink droplets (when required) through thermal or piezoelectric heads [78]. Using a piezoelectric inkjet printer controlled by a jetting voltage waveform, Boehm et al. fabricated a miconazole-loaded microneedle (Figure 3C) [123]. In addition, PolyJet printing is another additive manufacturing (AM) material jetting process in which liquid photopolymer droplets are deposited directly onto an elevator substrate [125]. The PolyJet process is particularly capable of using both stiff and flexible materials, and printing complex multi-material structures, by depositing two different materials on a pixel-by-pixel basis [125]. Using this PolyJet printing, flexible and biocompatible, bat-shaped 3D polymer structures can be fabricated, as shown in Figure 3D [126].

3D printing can create precise 3D structures using various types of materials and has been extended to biomedical applications. However, one disadvantage of 3D printing is that it only considers the primary state of the printed structures, which is static and inanimate [129]. Recently, four-dimensional (4D) printing technology has emerged to overcome this limitation [130,131]. 4D printing is based on the ability to change shapes or functions over time upon exposure to internal or external stimuli [130]. Owing to their simple manufacturing process, flexibility, and low cost, shape memory polymers (SMP) have been widely utilized as one of the main 4D printing materials [131]. Specifically, Ge et al. proposed a self-folding box by printing active SMP composites on hinges connected to inactive stiff panels (Figure 3E) [26]. They demonstrated that the box was thermally responsive to attain biaxially stretched open (heat-up) and closed (cool-down) states reversibly [26]. Recently, 4D printing technology has expanded by utilizing advanced material properties and systems to create more complex and multi-functional soft robots. Figure 3F demonstrates the movement of a 4D printed magnetic, hydrogel-based, octopus-shaped soft robot from left to right, corresponding to a programmed magnetic field [128]. Particularly, the octopus-shaped robot was printed by using dual hydrogels composed of acrylamide-carbomer (AAm-carbomer) and an AAm-carbomer-ferromagnetic particle (Fe_3_O_4_) bilayer. The bottom part of the robot is printed by using AAm-carbomer ink, while the upper part is printed by using AAm-carbomer ink mixed with magnetic particles. The octopus robot moves forward under the drive of a magnetic field programmed to move from left to right [128].

## 4. Applications of Biodegradable Soft Robots

### 4.1. Drug Delivery Carriers

**Figure 4:**
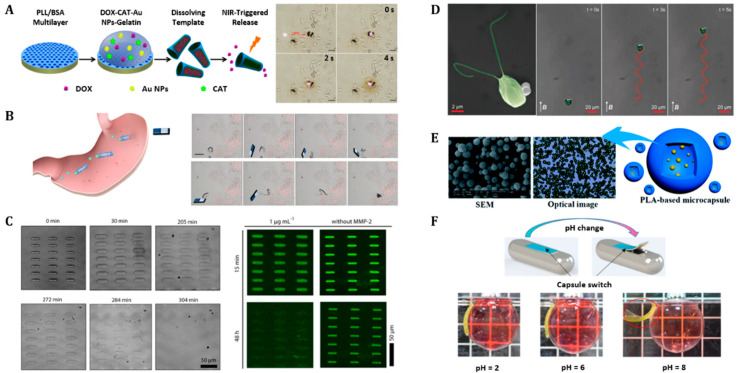
**Biodegradable drug delivery carriers.** (**A**) Fabrication and light-triggered drug release process of biodegradable (PLL/BSA)10-DOX-CAT-AuNPs-gelatin-based drug delivery rockets. Reproduced with permission [58]. Copyright 2015, The American Chemical Society. (**B**) Magnetic guidance of a single DOX/PASP/Fe-Zn MR in vitro for effective localization in the stomach. Reproduced with permission [90]. Copyright 2019, The American Chemical Society. (**C**) Microscale biodegradable swimmer array in the presence of an enzyme for drug release. Reproduced with permission [65]. Copyright 2019, The American Chemical Society. (**D**) Algal *(Chlamydomonas reinhardtii*) micro-swimmer’s propulsion trajectories under 26 mT of uniform magnetic field. Reproduced with permission [136]. Copyright 2018, Wiley-VCH. (**E**) SEM and optical images of a PLA-based microcapsule (left) and schematic image of the microcapsule (right). Reproduced with permission [75]. Copyright 2017, The Royal Society of Chemistry. (**F**) Actuator-controlled drug release model fabricated with a polyacrylic acid (PAAc) and polyacrylamide (PAAm) bilayer (left), and the PAAcPAAm bilayer soaked in different aqueous solutions of pH 2, 6, and 8 (right). Reproduced with permission [93]. Copyright 2017, The Royal Society of Chemistry.

Drug delivery is a method of administering drugs to achieve therapeutic effects in humans or animals [132]. Drug delivery studies have been directed toward continuously developing non-invasive, non-toxic, and safe-acting systems in humans [133]. Non-biodegradable drug delivery machines require removal via endoscopes or surgeries after releasing the drugs at the desired target areas [134]. To overcome this technical limitation, several biodegradable materials have been utilized to create biodegradable drug delivery machines that degrade automatically after a certain duration, without requiring any manual intervention [134,135]. Biodegradable drug delivery carriers have shown advanced functionalities in diverse forms of micro-rockets, micro-swimmers, and microcapsules [58,65,75,90,93,136,137]. For example, cylinder-shaped micro-rockets have been utilized as drug delivery carriers (Figure 4A,B) [58,90]. A micro-rocket is a microscale actuator, which can derive the fuel for its actuation from the human body (e.g., hydrogen peroxide (H_2_O_2_) [58] and gastric acid [90]). For specific examples, Figure 4A shows a micro-rocket composed of biodegradable bovine serum albumin (BSA) and poly-lysine (PLL) [58]. This micro-rocket is propelled by hydrogen peroxide (H_2_O_2_) and releases the drug, doxorubicin (DOX), at the desired site as a response to light in the near-infrared (NIR) region [58]. In addition, Figure 4B shows a micro-rocket manufactured using biodegradable poly(aspartic acid) (PASP) combined with a thin Fe intermediate layer and Zn core. This micro-rocket uses human gastric acid as its fuel for self-propulsion [90].

Micro-swimmers are micro/nanoscale devices with the ability to move in liquid environments [138]. One of the most commonly used forms is the helical-shaped micro-swimmer [32,64,65,139]. A helical microstructure can generate the required propulsive force by using an external rotating magnetic field (RMF) in a low-Reynolds-number environment [82]. This characteristic highlights the higher efficiency of magnetic torque compared to that of magnetic gradient pulling for microscale actions [140]. Therefore, helical micro-swimmers have received considerable attention for biomedical applications [32]. Figure 4C shows an example of a helical micro-swimmer composed of a biodegradable gelatin methacryloyl (GelMA) hydrogel for biomedical drug delivery and release at the target areas [65]. This GelMA-based micro-swimmer is combined with biofunctionalized superparamagnetic iron oxide nanoparticles for locomotion control via external magnetic guidance [65]. Biohybrid micro-swimmers that include organisms have been proposed as multi-functional and smart, small-scale soft robots [136,141]. Moreover, biohybrid systems enable the simultaneous achievement of advanced-level functions [141]. Besides the simple biohybrid system focusing on bacterial utilization for micro-swimmers [142,143,144,145,146], Yasa et al. proposed another advanced microalga-based biohybrid cargo delivery system (Figure 4D) [136]. This partially biodegradable, biohybrid micro-swimmer, comprised the unicellular, freshwater, green microalga, *Chlamydomonas reinhardtii*, and polyelectrolyte (PE)-functionalized 1 µm-diameter magnetic polystyrene (PS) particles [136]. The microalga-based biohybrid micro-swimmer exhibited high propulsion ability (>100 µm s^−1^), autofluorescence, and phototactic guidance capability [136].

Capsule-shaped carriers are also a type of drug delivery carrier used to encapsulate drugs and release them at targeted locations [75]. Drug delivery microcapsules must be encapsulated for long periods, and a sufficient durability and stability are essential. Poly(lactic acid) (PLA)-based capsules have been widely used because of their gradual degradation (Figure 4E) [75]. In addition, PLA microcapsules can be fabricated using electrospray (ES) [75] or lithography [137]. Another capsule-shaped drug delivery carrier composed of a spherical polyethylene structure with a polyacrylamide-polyacrylic acid (PAAm-PAAc) bilayer patch on top has been proposed (Figure 4F) [93]. PAAm (microbial-degradable [147]) and biodegradable PAAc [92] are pH-responsive hydrogels. When the pH < 6, the swelling ratio of PAAm is relatively large, whereas PAAc is dominantly expanded in a pH > 6 environment [93]. Thus, PAAc is attached to the capsule directly, while PAAm is attached to the capsule in the opposite direction, such that the bilayer bends in an alkaline environment [93].

### 4.2. Grippers

**Figure 5:**
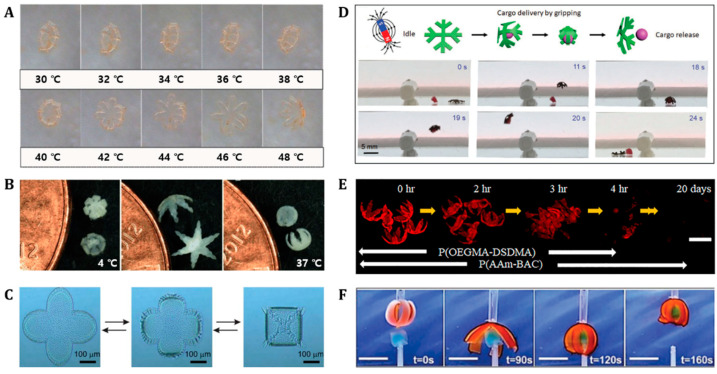
**Biodegradable grippers as actuators**. (**A**) Temperature-dependent unfolding of long Venus flytrap-like hydrogel grippers. Reproduced with permission [98]. Copyright 2014, Wiley-VCH. (**B**) Theragrippers originally closed at 4 °C, open as the temperature increased and finally closed again in the opposite direction at 37 °C. Reproduced with permission [88]. Copyright 2013, The American Chemical Society. (**C**) Self-folding of the thermoresponsive star-shaped bilayer gripper. Reproduced with permission [149]. Copyright 2013, The Royal Society of Chemistry. (**D**) Magnetic responsive gripper. Cargo grasping, transport, and release step under magnetic field gradients generated by a permanent magnet. Reproduced with permission [67]. Copyright 2020, Wiley-VCH. (**E**) Thermomagnetically responsive gripper at high 50 mM GSH. Reproduced with permission [135]. Copyright 2019, The American Chemical Society. (**F**) pH-responsive soft gripper loading an object in 0.1 M HCl aqueous solution (scale bars = 4 cm). Reproduced with permission [33]. Copyright 2017, The Royal Society of Chemistry.

Multiscale soft grippers have been significantly developed over the past few decades [148]. A variety of tethered and untethered soft grippers has been designed and controlled by the stimuli-on-off process. Stimuli-responsive soft grippers exhibit smart shape reconfigurations or movements such as pick-and-place, biopsy, and actuator tasks [5]. More recently, biodegradable, stimuli-responsive soft grippers demonstrated several multi-functional pick-and-place, biopsy, and actuating tasks under autonomously programmed thermal, magnetic, or light on-off triggers in unstructured aqueous environments (Figure 5) [33,67,88,98,135,149]—specifically, biodegradable poly(ethylene glycol) diacrylate (PEGDA), thermally responsive poly(*N*-isopropylacrylamide) (PNIPAM), and magnetic alginate composite gripper-encapsulated microbeads via the NIR laser irradiation on-off process, as demonstrated in Figure 5A [98]. The light-driven open and close actuation of the gripper has shown significant potential for targeted therapeutic drug delivery [98]. In addition, Figure 5B shows another thermoresponsive drug-loaded theragripper composed of biodegradable polypropylene fumarate (PPF) and a thermally responsive poly(N-isopropyl acrylamide-co-acrylic acid) (pNIPAM-AAc) bilayer [88]. Responding to temperature changes, at 4 °C, the closed theragripper opens its hands gradually as the temperature increases and closes in the opposite direction when the temperature reaches 37 °C (i.e., physiological body temperature) [88]. Another partially biodegradable and thermoresponsive, star-shaped poly(NIPAM-ABP)/ polycaprolactone (PCL) bilayer gripper is shown in Figure 5C [149]. Responding to low temperatures (T < 10 °C), the poly(NIPAM-ABP) layer swells and the gripper folds; as the temperature increases, the poly(NIPAM-ABP) layer shrinks and the gripper unfolds. The gripping and releasing motions of the gripper are completely reversible in response to the temperature signal. Moreover, the gripper exhibits different folding temperatures and degradation rates depending on the thickness of each layer [149].

In addition to thermally responsive biodegradable grippers, various magnetically responsive biodegradable grippers have been proposed [67,135]. The use of magnetic nanoparticles allows the gripper to deform its shape and actuate in response to an applied magnetic field. For example, a magnetically responsive, biodegradable, collagen-based hydrogel milli-gripper, with embedded superparamagnetic iron oxide nanoparticles (SPIONs), is shown in Figure 5D [67]. By modulating the magnetic field within the 5–25 mT range, both the folding and movement of the gripper was controlled. After completing a pick-and-place task via a magnetic field on-off process, the gripper completely biodegraded using the matrix metalloproteinase-2 enzyme [67]. Another magnetically guided and thermally actuated, biodegradable soft gripper is shown in Figure 5E [135]. The gripper consists of thermally responsive high-swelling poly(oligoethylene glycol methyl ether methacrylate (Mn = 500)-bis(2-methacryloyl)oxyethyl disulfide) (P(OEGMA-DSDMA)) and low-swelling poly(acrylamide-*N*,*N*′-bis(acryloyl)cystamine) (P(AAm-BAC)) gels doped with Fe_2_O_3_ nanoparticles. Owing to the difference in the swelling rate of each layer, the shape of the gripper transformed within a 50–70 °C temperature range. The P(OEGMA-DSDMA) layer degraded completely in 4 h, while the P(AAm-BAC) layer degraded in 20 days in an acidic environment (pH = 3) [135]. Furthermore, a pH-responsive biodegradable soft chitosan and carboxymethylcellulose (CMC) bilayer grippers have been introduced (Figure 5F) [33]. Chitosan and CMC exhibit high swelling in low pH and high pH environments, respectively. Using the characteristics of the different pH responses of chitosan and cellulose/CMC, the gripper is opened and closed reversibly in response to different pH environments. In a 0.1 M HCl aqueous solution, the arms of the gripper were bent to grip the target. Subsequently, the gripper lifted the cargo and opened its arms to release it in a 0.1 M NaOH solution [33].

### 4.3. Tissue Engineering

**Figure 6:**
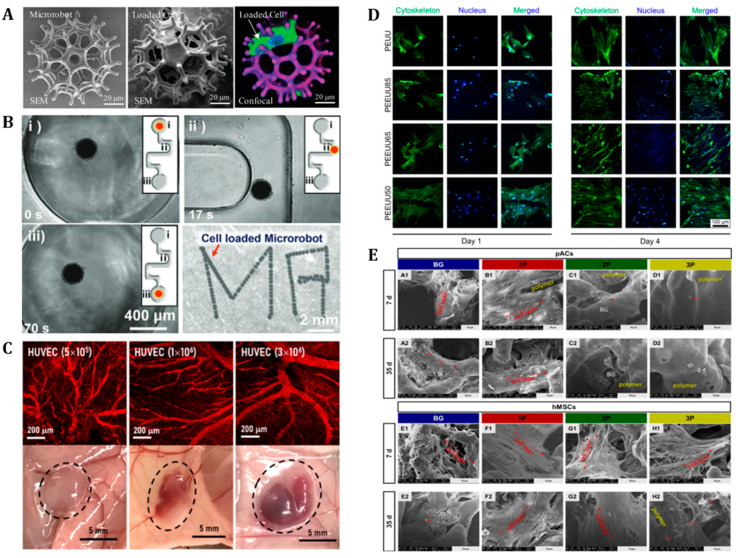
**Biodegradable tissue engineering.** (**A**) SEM images of a PEGDA-PETA microrobot structure (left), cell-loaded microrobot (middle), and confocal scan of green fluorescent protein (GFP)-labeled MSCs (GFP-MSCs) cultured on the microrobot (right). Reproduced with permission [152]. Copyright 2020, Wiley-VCH. (**B**) Magnetic actuation of the microrobot in a confined microfluidic channel (**i**–**iii**), and the magnetic manipulation of the GelMA microrobot to write “MR”. Reproduced with permission [77]. Copyright 2022, Wiley-VCH. (**C**) Representative CLSM images of DFO (0.1%) and HUVEC (5 × 10^5^, 1 × 10^6^, and 3 × 10^6^)-laden P5L1.1 gels (nanocomposite gels containing 5.0% (*w*/*v*) PLGA-PEG-PLGA and 1.1% (*w*/*v*) laponite), formed in the subcutaneous tissue of mice. Reproduced with permission [80]. Copyright 2022, The American Chemical Society. (**D**) Immunofluorescence images of vSMCs cultured on PEUU, PEEUU85, PEEUU65, and PEEUU5 with the labeling of the cytoskeleton (green) and nucleus (blue) after 1 and 4 days, respectively. Reproduced with permission [153]. Copyright 2022, The American Chemical Society. (**E**) SEM images of colonized, bioactive glass-based scaffolds. BG, 1P, 2P, and 3P mean the pure bioactive glass, single, twofold, and threefold PLGA infiltrations. Reproduced with permission [160]. Copyright 2022, MDPI.

Tissue engineering incorporates biology into engineering to create or repair tissue or cell products either in vitro or in vivo [150]. One of the ultimate goals of tissue engineering is to improve or replace biological tissues. The biocompatible scaffold to which stem cells are attached must be moved to the target body area to allow the stem cells to settle in that area, after which the scaffold must be biodegraded. For this operation, the chemistry, porosity, and biodegradability must be adjusted according to the biomaterial required for scaffold fabrication [151]. Biodegradable soft robots have been extensively utilized in tissue engineering. For example, Figure 6A shows a burr-like, porous, spherical micro-swimmer loaded with mesenchymal stem cells (MSCs) in its pores [152]. This micro-swimmer is composed of biodegradable poly (ethylene glycol) diacrylate (PEGDA) and pentaerythritol triacrylate (PETA). Since this tissue engineering system has a burr-like spherical geometry, the number of cells loaded is greater than that of a typical porous spherical structure (such as in advanced cancer therapeutic soft robots) [152]. Figure 6B shows another biodegradable microrobot for stem cell delivery [77]. This microrobot was fabricated using gelatin methacrylate (GelMA) and superparamagnetic iron oxide nanoparticles (SPIONs), Fe_3_O_4_, owing to their biodegradability, biocompatibility, and magnetic-based cell delivery system. Previous research regarding GelMA microrobots chose conventional fabrication methods, such as two-photon polymerization. Two-photon polymerization is capable of manufacturing sophisticated micro- or nanorobotics; however, it has a long fabrication cycle for a single microrobot, so mass producing diverse applications is difficult. Furthermore, resins containing magnetic nanoparticles (MNPs) are hard to polymerize during the laser writing process. Reducing the number of MNPs is inappropriate to overcome the polymerization limitation because a small number of MNPs may cause ineffective manipulation of the robot. To make the mass production of GelMA microrobots possible as well as maintaining the proper amount of MNPs, Noh et al. selected a microfluidic channel mass production method to manufacture GelMA microrobots. Human nasal turbinate stem cells (hNTSCs) were cultured in this GelMA microrobot. It was precisely controlled to reach the target area via an external rotating magnetic field, upon which the hNTSCs were finally released into the neuronal cells [77].

Another scaffold loaded with stem cells and drugs is shown in Figure 6C [80]. Specifically, desferrioxamine (DFO) and human umbilical vein endothelial cells (HUVECs) were combined with biodegradable poly(DL-lactide-co-glycolide)-b-polyethylene glycol-b-poly(DL-lactide-co-glycolide) (PLGA-PEG-PLGA) to create smart scaffolds to promote vascularization in in vivo tissue engineering applications [80]. Additionally, Figure 6D describes another human umbilical arterial smooth muscle cells (vSMCs)-loaded, biodegradable, hydrogel-based scaffold, which can be utilized to effectively repair tissue defects via tissue engineering [153]. The cell-loaded scaffold was fabricated using poly(ester-ether-urethane)ureas (PEEUUs), polyurethane (PU)-based polymers, synthesized through a two-step solution polymerization using polycaprolactone (PCL) diol and polyethylene glycol (PEG). Owing to its significant characteristics of biodegradability and biocompatibility [153], PU has been widely used in tissue engineering [154,155,156,157]. The majority of current research studies related to biodegradable PU scaffolds have focused on adjusting their chemical and mechanical properties at the molecular level to enhance the geometric stability and biocompatibility of the scaffolds [153,158,159].

Figure 6E describes a poly(D-L-lactide-co-glycolide) (PLGA)-infiltrated bioactive glass scaffold cultivated with human mesenchymal cells (hMSCs) for cartilage regeneration [160]. Silicon dioxide or silicate-based bioactive glasses are nonporous, bioceramic, hard material comprising three basic components (e.g., sodium dioxide, calcium oxide, and phosphorous) [161]. This bioactive glass has mainly been used for bone regeneration and has recently been extended to various tissue engineering fields [162,163,164,165]. Bioactive glass was developed to provide cells with the ability to adhere, survive, and proliferate, but was too brittle to endure the mechanical load of the human knee joint [160]. Unlike conventional bioactive glass composite scaffolds [166,167], biodegradable and biocompatible PLGA-infiltrated bioactive glass can improve scaffold stability and biocompatibility by using PLGA infiltration [160,168]. This scaffold maintained a stable shape and performed a decent cell culture even in a 35-day cell cultivation process without showing any degradation. Although PLGA is a biodegradable material [168], it has a relatively long biodegradation period of at least 18 months [160].

## 5. Conclusions and Outlook

In summary, significant advances in stimuli-responsive biodegradable soft robots were discussed in terms of their design, fabrication, and application of biodegradable materials. A variety of biodegradable materials have shown extensive potential in biomedical applications such as multi-functional drug delivery carriers, grippers, and tissue engineering. To create complex, biodegradable 3D soft robots for biomedical applications, highly precise 3D fabrication methods have been developed along with advances in biodegradable material synthesis strategies. Owing to their scalability and manufacturability, photolithographic and 3D/4D printing methods have been preferentially adapted over the past few decades to develop multiscale and multi-functional 3D soft robots.

Despite the significant development of stimuli-responsive, biodegradable soft robots, most of them remain in the conceptual stages. First, naturally synthesized biodegradable materials have superior biocompatibility; however, their poor mechanical properties limit their wide range of application. In addition, artificially synthesized biodegradable materials can provide more improved mechanical properties than natural biodegradable matters. Nevertheless, most of them are sensitive to temperatures, solvents, or water, such that they pose other challenges to selecting suitable fabrication strategies [169]. More recently, smart, hybrid, biodegradable materials have shown another possibility to developing multi-functional, intelligent, soft robots in the near future [170,171]. In addition, to overcome the limitations of biodegradable soft robots, precise and selective magnetic, electric, thermal, or pH control systems have accompanied the developments of biodegradable material syntheses and high-throughput fabrication methodologies. Furthermore, biodegradable stimuli-responsive soft robots have rarely been explored in real in vivo environments for intelligent clinical drug delivery, biopsy, or tissue engineering. To successfully develop real in vivo models, the autonomous and precise navigation and manipulation of stimuli-responsive, biodegradable soft robots must be confirmed in the near future. In conclusion, the new perspective of smart, biodegradable soft robots has aligned well with all the developments in multidisciplinary materials science, and the mechanical, electrical, and biomedical engineering fields.

## Figures and Tables

**Table 1:** Pros and cons of biodegradable materials and their applications.

Type	Material	Advantage	Disadvantage	Application
Natural polymer	Chitosan	Enzymatically degraded by lysozyme and chitosanase enzymes [35]	Water-insoluble, unstable, toxic at hydrogel phase [70]	Targeted drug/cell delivery [32] Gripper [33]
	Cellulose/carboxymethylcellulose (CMC)	Biocompatible, soft, transparency, high viscosity at low concentrations, swelling at high pH [32,37]	Weak mechanical properties [71]	Gripper [33]
	Gelatin	Low gelation temperature: 22.4–25.2 °C [38,39] Non-toxic, high water absorption, biocompatible [40,41,42]	Weak mechanical properties [72]	Tissue engineering [73] Drug delivery [74]
Synthetic polymer	Poly(lactic acid) (PLA)	Degraded by the hydrolysis of ester bonds without requiring any enzymes [44]	Slow degradation rate, hydrophobicity, low impact toughness [44]	Drug delivery [75] Surgical implant [76] Tissue engineering [76]
	Poly-L-lysine (PLL)	Hydrophilic, biocompatible [56]	Cytotoxicity increases with its molecular weight [62]	Gene delivery [59,62]
	Gelatin methacryloyl (GelMA)	Degraded by cell-released enzymes [64]	Low mechanical strength (~50 to 150 KPa), short degradation time (~7 to 14 days) [63]	Drug delivery [64,65] Tissue engineering [69,77]
	Poly(ethylene glycol) (PEG)	Non-ionic, low inflammation [78]	Low mechanical strength [79]	Tissue engineering [80]
	Poly(ethylene glycol) diacrylate (PEGDA)	Mechanical stability [79]	Slow degradation rate in vivo [81]	Drug delivery [82] Tissue engineering [68]
	Poly(propylene fumarate) (PPF)	Biocompatible, non-toxic [83,84,85,86,87]	Mechanical strength loss, brittleness during degradation [83]	Gripper [88,89]
	Poly(aspartic acid) (PASP)	Smooth, intact, robust [90]	Complex synthesis [91]	Drug delivery [90] Tissue engineering [91]
	Poly(acrylic acid) (PAAc)	Water-soluble, high molecular-weight, pH-responsive [92]	Low mechanical strength [92]	Drug delivery [93]
	Poly(ε-caprolactone) (PCL)	Semi-rigid at room temperature [94]	Slow degradation rate, low stiffness [95]	Tissue engineering [96]

## Data Availability

The data presented in this study are available on request from the corresponding author.
